# Enhanced digestion of complex cosmetic matrices for analysis of As, Hg, Cd, Cr, Ni, and Pb using triton X-100

**DOI:** 10.1016/j.mex.2021.101241

**Published:** 2021-01-23

**Authors:** Devika Maharaj, Terry Mohammed, Azad Mohammed, Letetia Addison

**Affiliations:** a Department of Chemistry, Faculty of Science and Technology, The University of the West Indies, St. Augustine, Trinidad and Tobago; b Department of Life Sciences, Faculty of Science and Technology, The University of the West Indies, St. Augustine, Trinidad and Tobago; c The University of the West Indies, St. Augustine, Trinidad and Tobago

**Keywords:** Heavy metal analysis, Cosmetic analysis, Acid Digestion, Heavy Metals, Cosmetic, Digestion

## Abstract

A suitable optimized digestion method for lipsticks and powders for the analysis of As, Hg, Cd, Cr, Ni and Pb by Hydride Generation Atomic Absorption Spectrophotometry (HG-AAS), Cold Vapor Atomic Absorption Spectrophotometry (CV-AAS) and Flame Atomic Absorption Spectrophotometry (FAAS) was developed using common acid digestion methods enhanced by the use of Triton X-100. The three acid digestion methods used in this study were Method A (nitric acid and hydrogen peroxide), Method B (nitric acid and hydrochloric acid) and Method C (nitric acid, hydrochloric acid and hydrogen peroxide). Triton X-100 was added to each of these and the effects were studied. The acid digestion method that was determined to be the most suitable and efficient for lipsticks and powders was Method A-1 (nitric acid and hydrogen peroxide with 5% Triton X-100 at 95 °C for 3 h). The range of percentage recoveries obtained were; powders (98.50% to 92. 61%) and lipsticks (100.96% to 99.41%) for As, Cd, Cr, Pb, Hg and Ni. The addition of Triton X-100 significantly improved the efficiency of the method.

• Triton X-100 improves the efficiency of acid digestion of fatty hydrophobic samples by dispersing the sample throughout the acid digestant.

## Graphical abstract

**Figure:**
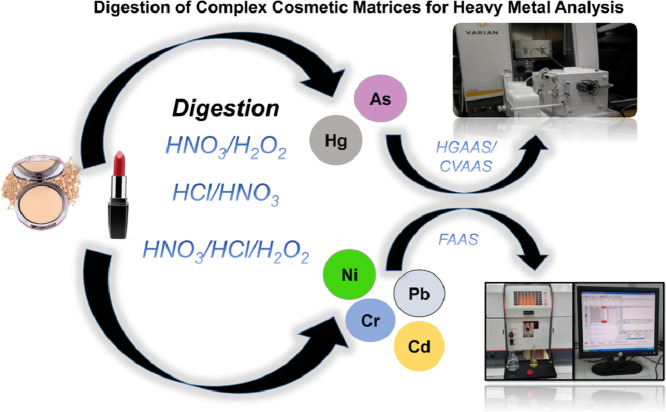

**Specifications Table**

**Table:** 

Subject Area:	
More specific subject area:	*Analytical Chemistry, Cosmetic Analysis*
Method name:	*Analysis of Heavy Metals in Cosmetics*
Name and reference of original method:	A. Khalid, I.H. Bukhari, M. Riaz, G. Rehman, Q. Ain, T.H. Bokhari, N. Rasool, M. Zubair, S. Munir, Determination of lead, cadmium, chromium, and nickel in different brands of lipsticks, International Journal of Biology, Pharmacy and Allied Sciences 1(2) (2013) 263–271.
Resource availability:	*NA*

**Method details**

## Method selection

Cosmetics are among the least regulated items imported into many developing countries. Being neither a Food nor a Drug, this commodity finds itself immune to many country's regulations, particularly developing nations. The analysis of cosmetics has only recently found itself on the mind of regulators as more and more evidence show the potential health risk of daily use of some cosmetics. Analytical techniques must therefore be fast, accurate, reproducible and low cost to meet the needs of developing nations.

A review of available literature reveals that digestion techniques used for the analysis of Heavy Metals in Cosmetics vary widely. Both microwave and Open Digestion are common, but the array of acid combinations coupled with digestion times, temperatures and programs vary widely. From available literature, it can be difficult to determine the optimal digestion combination. The challenge is further complicated by the matrix of the sample to be digested. Cosmetic samples tend to possess highly variable matrices from inorganic powders to waxy lipsticks, and establishing a single harmonized digestion procedure will allow for efficient and large-scale analysis of multiple cosmetic sample matrices. Table 1 summarises some of the more common analytical techniques used to digest cosmetic matrices.

**Table 1:** Common digestion regimes for cosmetics.

Cosmetic Type(s)	Acid(s) and Digestion Type	Time and Temperature	Metals Detected	Instrument	Author(s)
Lipsticks	Microwave Digestion 6 mL of 69% HNO_3(aq)_	15 min at 130 °C, 15 min at 200 °C 10 min at 50 °C	Pb, Cd and Cr	ICP-OES	(Zakaria and Ho, 2015) [1]
Eye Shadows	Open Digestion 5 mL, 2 M HNO_3(aq)_, 2 mL 30% H_2_O_2(aq)_ 1 mL 5% Triton X-100	3 hrs, 100 °C	Cd, Co, Cr, Cu, Ni, and Pb	GFAAS	(Batista et al., 2014) [2]
Lipsticks	Wet Digestion HNO_3(aq)_ and H_2_O_2(aq_	<100 °C until fuming	Pb, Cd, Cr, Ni	FAAS	(Khalid et al., 2013) [3]
Creams	Open Digestion 5 mL of 65% HNO_3(aq)_ and 70–72% HClO_4(aq)_	2–3 hrs on hot plate	Al, Cu, Mn, Pb, Cr, Co, Ni, Cd, As, Hg	ICP-MS	(Salama, 2016) [4]
Powders, creams, lipsticks	Open Digestion 4:1 mixture 65% HNO_3(aq)_ : 70% HClO_4(aq)_	3 hrs	Cd, Cr, Pb, Mn, Ni, Cu	FAAS	(Sani et al., 2016) [5]
Creams, powders, eye liners	Open Digestion 10 mL Aqua Regia (HNO_3(aq)_ and HCl_(aq)_)	30 min at 150 °C	Cd, Zn, Pb, Ni	FAAS	(Omenka and Adeyi, 2016) [6]
Lipstick, Eye shadows, powders, mascaras	Open Digestion 20 mL HNO_3(aq),_ 10 mL HCl_(aq)_) 5 mL H_2_O_2(aq)_	2 hrs at 125 ^o^C	Cd, Pb, Ni, Cr, Co, Fe, Mn, Zn,	FAAS	(Iwegbue et al., 2016) [7]

The quantity of heavy metals extracted from a sample depends entirely upon the sample preparation procedure and experimental conditions used [8]. Several digestion methods such as dry ash, wet acid, and microwave acid digestion are used in the decomposition of a sample. Of the digestion methods available, microwave digestion is the most efficient and appropriate method to solubilize biological samples [9], however, it requires an expensive digester [10] and it has relatively low productivity [11]. Dry ash digestions are not labor exhaustive, they require few reagents and numerous samples may be analyzed simultaneously, however, muffle furnaces are expensive, extensive digestion time is required and loss of volatile metals due to high temperature are common challenges of this procedure [12,13].

Open digestion, specifically open acid digestion, presents the most feasible option for this study since it is effective on both organic and inorganic materials [14] and there is minimum loss of volatile metals due to lower temperatures when compared to the temperatures of dry ash digestion [12,14]. Open digestion is inexpensive and the simple control of important parameters such as reagents, time and temperature makes it a simple and reliable tool for routine analysis. However, this system is limited by low utmost digestion temperature, hence; the temperature is unable to surpass the boiling point of the acid or acid combination mixture used. It is therefore important to include quality control measures such as certified reference materials (CRM) or spike samples followed by percent recoveries as routine elements of the analysis.

The metals being analyzed and the sample matrix are important factors in selecting the most efficient digestion reagents and conditions. Nitric acid is considered a universal decomposition reagent since it does not interfere with most determinations [15], also a combination of nitric acid, hydrogen peroxide and hydrochloric acid can be conveniently used to improve the quality of decomposition. Combinations with hydrofluoric acid are also utilized, however, safety precautions are crucial [16]. Various researchers used nitric acid, hydrochloric acid, hydrofluoric acid, perchloric acid and hydrogen peroxide individually or in combinations [1,^5^,^7^,[17], [18], [19], [20], [21], since there exist no specific acid or acid combinations for the analysis of heavy metals in cosmetics.

Digestion temperature and digestion time are equally important as it determines the effectiveness of a digestion. High temperatures are sometimes required to attain a complete decomposition [15], however, high temperature can result in loss of volatile metals [14] such as lead and mercury. The digestion time controls the length of exposure of the matrix to the digestion reagents. Loss of volatile metals can further occur since the exothermic digestion process is increased due to the length of exposure [14]. One to two hours are the usual open acid digestion times used for decomposition of a typical sample [22], however, shorter or longer times can be employed. Too short a digestion period can result in incomplete digestion whereas too long a digestion time can result in loss due to volatilization. It is therefore crucial to select the most effective time and temperature to attain the highest yield of the metal.

Another factor that must be considered for acid digestion is contact between sample and digestant. For aqueous and acid miscible liquids, matrix and acid contact is maximized. For solids, particle size reduction is recommended to increase the surface area for contact between matrix and digestant [23]. For semi-solid immiscible matrices such as lipsticks, surface area contact is minimal and acid digestion tends to be poor. Typically for these types of samples, the matrix is destroyed in a furnace at high temperatures at the expense of volatile metals [13]. However, to facilitate digestion by acids, a surfactant can be used to disperse the immiscible solid within the acid solution [2].

The methods of Khalid et al. [3] , Omenka and Adeyi [3,6] and Iwegbue et al. [7] were evaluated with and without the use of a surfactant. Triton X-100 was incorporated due to its homogenization nature. Triton X-100 is a non-ionic surfactant and can be used to prevent small particles such as powders from adhering to the sides of glassware due to surface water tension [2].

## Materials and methodology

### Instrumentation

Cosmetic samples were analyzed for Cd, Cr, Pb, and Ni using the Spectra AA 880 Atomic Absorption Spectrophotometer (Varian Inc. USA) and for As and Hg using the Agilent Vapor Generation Accessory VGA-77, coupled with the SpectrAA 880 Atomic Absorption Spectrophotometer (Agilent Technologies Inc. USA), using the method of Mohammed et al. [24].

### Materials

The following material were used in this study:

• Nitric Acid, ACS Grade 70% (Sigma-Aldrich, USA)
• Hydrochloric Acid, ACS Grade 37% (Sigma-Aldrich, USA)
• Hydrogen Peroxide, ACS Grade 30% (Sigma-Aldrich, USA)
• Triton-X 100, ACS Grade (Sigma-Aldrich, USA).
• 50 mL Boiling Tubes (Pyrex, USA)
• Whatman 541 Hardened Ash less Filter Paper (Sigma-Aldrich, USA)
• Class A 50 mL and 25 mL Volumetric Flasks (Pyrex, USA).
• 1000 µg/mL Stock Solutions As, Cd, Cr, Pb, Hg and Ni (Accustandard, USA
• Tin (II) chloride, ACS Grade (Sigma-Aldrich, USA)
• Sodium Borohydride, ACS Grade (Sigma-Aldrich, USA)
• Potassium Iodide, ACS Grade (Sigma-Aldrich, USA).

### Glassware preparation

All glassware were washed with an anionic detergent and rinsed with tap water followed by deionized water. They were then soaked for 24 h in a diluted nitric acid bath, and then rinsed with deionized water. The glassware were air dried at room before use.

### Sample selection

A mineral face powder and a lipstick sample were used in this study. These were selected as they represented the two extremes of the cosmetic matrices; Powder being primarily inorganic and finely ground and lipstick being heavy and waxy.

### Sample preparation

0.5 ± 0.1 g of face powder and lipstick samples were weighed in triplicate into clean, dried labelled boiling tubes. Reagents were added to the samples as illustrated in Table 2. The samples were then pre-digested at room temperature for 24 h then digested on a heating block at the appropriate times and temperatures as defined in Table 2. Samples were then cooled to room temperature and filtered through No. 541 Whatman filter paper into 50 mL volumetric flasks. The filtrate made up to the mark with deionized water. This was used for the analysis of Cd, Cr, Hg, Pb and Ni.

**Table 2:** Acid digestion regimes used in this study.

		*Acid Combinations*	*Volume (mL)*	*Temperature (*°*C)*	*Time (hours)*
With Triton-X 100, 5% 1 mL	A-1	70% Nitric Acid 30% Hydrogen Peroxide	5 5	95	3
	B-1	37% Hydrochloric acid 70% Nitric Acid	7.5 5	108	2
	C-1	70% Nitric Acid 37% Hydrochloric Acid 30% Hydrogen Peroxide	8 4 2	125	2
Without Triton-X 100	A-2	70% Nitric Acid 30% Hydrogen Peroxide	5 5	95	3
	B-2	37% Hydrochloric acid 70% Nitric Acid	7.5 5	108	2
	C-2	70% Nitric Acid 37% Hydrochloric Acid 30% Hydrogen Peroxide	8 4 2	125	2

For Arsenic analysis, reduction of Arsenic (V) to Arsenic (III) is necessary for the formation of the stable hydride needed for HGAAS. In order to achieve this, 5 mL aliquots were removed from the filtrate and combined with 5 mL of 1% potassium iodide in 1 M HCl in a boiling tube. The digests were left to reduce for 50 min at room temperature, then filtered through a No. 541 Whatman filter paper into 25 mL volumetric flasks, after which they were made up to 25 mL with deionized water.

### Quality assurance

Analytical blanks were prepared and analyzed for each digestion regime in a manner identical to the samples. The blank values were subtracted from all analytical results.

Precision was expressed by the Relative Standard Deviation (RSD) or Standard Deviation of the Mean (SDM) [25]. In this study a RSD value of ≤ 5% was considered acceptable. The RSD was calculated using the following formula:

(1) $$RSD=\frac{Standard\;Deviation}{Mean}\;\times \;100$$

Certified Reference Materials (CRM) for cosmetics were unavailable, therefore, spiked samples were prepared for each of the digestion regimes to give a final spike concentration of 1 ppm of Cd, Cr, Pb and Ni, and 50 ppb of As and Hg. The spiked samples were analyzed and percent recovery were calculated. Acceptable recoveries typically range from 80 to 120 percent [26]. The percentage recovery was calculated using the following equation [27]:

(2) $$Recovery\;\%\;=\frac{\left(Spiked\;Concentration\;Sample\;--\;Unspiked\;Concentration\;Sample\right)\;}{Concentration\;added}\;\times 100$$

To measure the quality of the correlation between absorbance and concentration values in the calibration curves used, the correlation coefficient (R^2^) was utilized. A R^2^ value ranging from 0.9950 to 0.9999 was deemed acceptable and a good fit for purpose [27]. Calibration curves having R^2^ values external to this range were rejected.

The Limit of Detection (LOD) was calculated using the following equation [27], [28], [29]:

(3) $$LOD=\frac{3s}{m}$$

## Method evaluation and validation

The concentrations obtained for the lipstick and face powder samples are shown in Table 3 and Table 5 respectively. For both lipstick and powder samples, the RSD% were below 5% which indicated that the values were close to the average value. As shown in Table 3, the average concentration of As, Cd, Cr, Pb, Hg and Ni using Method A-1 were higher than the average concentrations obtained from Methods B-1, C-1, A-2, B-2 and C-2. Method A-1 extracted all of the heavy metals of interest from the lipstick samples and minimized the loss of the heavy metals. The statistical comparison displayed in Table 4 showed that the average concentration for the heavy metals of interest in lipsticks digested with the addition of Triton X-100 was slightly higher (5.60 ± 12.92 mg/kg) than that recorded without the use of Triton X-100 (5.22 ± 13.62 mg/kg). In both cases, the minimum concentrations were 0.00 mg/kg, while maximum concentrations were 50.52 mg/kg (With Triton X-100) and 56.86 mg/kg (without Triton X-100). The 95% Confidence Interval suggests that is a large number of samples if taken, 95% of the time the average concentration will falls within the interval ( −0.82, 12.03 mg/kg) with Triton X-100 and (−1.61, 12.06 mg/kg) without Triton X-100.

**Table 3:** Average Concentration (mg/Kg) ± RSD of lipsticks using different acid digestion methods.

	Elements	Acid Digestion Methods		
		A-1 (HNO_3(aq)_ + H_2_O_2(aq)_)@ 95 °C for 3 h	B-1 (HCl_(aq)_ + HNO_3(aq)_@108 °C for 2 h	C-1 (HNO_3(aq)_ + HCl_(aq)_ + H_2_O_2(aq)_) @ 125 °C for 2 h
With Triton X-100	As	0.93 ± 2.85	0.59 ± 0.81	0.72 ± 3.96
	Cd	0.57 ± 0.87	0.48 ± 2.77	0.50 ± 1.73
	Cr	4.59 ± 1.24	<LOD_Cr_*	<LOD_Cr_*
	Pb	4.55 ± 3.28	0.79 ± 0.51	1.96 ± 1.41
	Hg	0.06 ± 1.31	0.03 ± 3.88	0.06 ± 0.66
	Ni	50.52 ± 0.22	7.16 ± 2.87	27.37 ± 1.79

		A-2 (HNO_3(aq)_ + H_2_O_2(aq)_)	B-2 (HCl_(aq)_ + HNO_3(aq)_	C-2 (HNO_3(aq)_ + HCl_(aq)_ H_2_O_2(aq)_)

Without Triton X-100	As	0.72 ± 0.19	0.22 ± 2.13	<LOD_As_⁎⁎
	Cd	0.42 ± 3.34	0.18 ± 0.04	0.29 ± 3.77
	Cr	2.41 ± 3.56	<LOD_Cr_*	<LOD_Cr_*
	Pb	2.79 ± 2.97	0.66 ± 0.04	3.39 ± 3.97
	Hg	0.04 ± 2.81	0.02 ± 2.96	0.05 ± 2.50
	Ni	56.86± 0.18	5.55 ± 1.41	20.37 ± 1.27

⁎ **LOD _Cr_ = 7.09 x 10^−2^** **mg/L**.

⁎⁎ **LOD _As_ = 1.99 x 10^−4^** **mg/kg**.

**Table 4:** Statistical analysis of Heavy Metal data derived from Lipsticks analyzed using Triton x100 and without Triton x-100.

	N	Mean	Std. Deviation	Std. Error	95% Confidence Interval for Mean		Minimum	Maximum
					Lower Bound	Upper Bound		
With Triton x-1000	18	5.60	12.92	3.04	−0.8207	12.0260	0.0000	50.5238
Without Triton x-100	18	5.22	13.75	3.24	−1.6141	12.0617	0.0000	56.8612

**Table 5:** Average Concentration (mg/kg) ± RSD of face powders using different acid digestion methods.

	Elements	Acid Digestion Methods		
		A-1 (HNO_3(aq)_ + H_2_O_2(aq)_)@ 95 °C for 3 h	B-1 (HCl_(aq)_ + HNO_3(aq)_@108 °C for 2 h	C-1 (HNO_3(aq)_ + HCl_(aq)_ + H_2_O_2(aq)_) @ 125 °C for 2 h
With Triton X-100	As	0.75 ± 2.75	0.66 ± 0.10	<LOD_As_**
	Cd	1.46 ± 1.03	1.39 ± 2.86	0.90 ± 1.79
	Cr	<LOD_Cr_*	<LOD_Cr_*	2.6594 ± 3.62
	Pb	10.79 ± 0.11	9.62 ± 1.22	10.28 ± 0.92
	Hg	0.06 ± 1.42	0.03 ± 3.11	0.07 ± 3.75
	Ni	93.09 ± 0.02	34.44 ± 1.46	93.55 ± 0.17

		A-2 (HNO_3(aq)_ + H_2_O_2(aq)_)	B-2 (HCl_(aq)_ + HNO_3(aq)_	C-2 (HNO_3(aq)_ + HCl_(aq)_ H_2_O_2(aq)_)

Without Triton X-100	As	0.68 ± 0.61	0.45 ± 0.28	<LOD_As_⁎⁎
	Cd	1.04 ± 3.09	0.64 ± 0.67	0.53 ± 2.98
	Cr	<LOD_Cr_*	<LOD_Cr_*	1.23 ± 0.03
	Pb	8.11 ± 2.31	8.62 ± 2.54	1.48 ± 0.03
	Hg	0.04 ± 3.69	0.04 ± 1.64	0.05 ± 0.03
	Ni	61.08 ± 0.43	21.77 ± 1.17	5.03 ± 0.05

⁎ **LOD _Cr_ = 7.09 x 10^−2^** **mg/L**.

⁎⁎ **LOD _As_ = 1.99 x 10^−4^** **mg/L**.

Method A-1 average concentrations for powders were higher than B-1, A-2, B-2 and C-1 average concentrations, however, Method C-1 average concentrations for Cr, Hg and Ni were higher than Method A-1. Method A-1 extracted the maximum concentration from As, Cd and Pb from the powdered samples while Method C-1 extracted the maximum concentration from Cr, Hg and Ni. Though Method C-1 was the only method that extracted Cr in powders, the average concentrations for Hg and Ni were slightly higher than that of Method A-1. The statistical comparison displayed in Table 6 showed that the average concentrations for the heavy metals of interest in powders digested with the addition of Triton X-100 were higher (14.43 ± 29.87 mg/kg) than that recorded without the use of Triton X-100 (6.15 ± 14.74 mg/kg). In both cases, the minimum concentrations were 0.00 mg/kg, while maximum concentrations were 93.54 mg/kg (With Triton X-100) and 61.09 mg/kg (without Triton X-100). From this, it was evident that the methods that incorporated the use of Triton X-100 extracted the highest heavy metal concentrations. The 95% Confidence Interval suggests that if a large number of samples are taken, 95% of the time the average concentration will fall within the interval ( −0.41, 29.29 mg/kg) with Triton X-100 and (−1.74, 13.48 mg/kg) without Triton X-100.

**Table 6:** Statistical analysis of Heavy Metal data derived from Face Powders analyzed using Triton x100 and without Triton x-100.

	N	Mean	Std. Deviation	Std. Error	95% Confidence Interval for Mean		Minimum	Maximum
					Lower Bound	Upper Bound		
With Triton x-100	18	14.4366	29.8734	7.0412	−0.4191	29.2923	0.0000	93.5482
Without Triton x-100	18	6.1574	14.7436	3.4751	−1.1744	13.4892	0.0000	61.0900

In the absence of a certified reference material, the percentage recovery of spiked samples were evaluated for samples digested with and without Triton x-100 (Tables 7 and 11). In Table 10, the Analysis of Variances (ANOVA) test suggests that, at the 5% level, there are statistically significant differences among the mean recoveries of the lipsticks using the different digestion methods, since *p* = 0.001 (i.e. *p* < 0.05). In this test, the assumption of equal population variance is met by the data as shown in Table 11, since *p* = 0.489 (i.e., *p* > 0.05). The statistical comparison as displayed in Table 8 for lipsticks, showed that the percentage recovery for the heavy metals of interest digested with the addition of Triton X-100 were higher (88.24 ± 13.33%) than that recorded without the use of Triton X-100 (80.42 ± 11.94%). The minimum recoveries were 60.90% (with Triton X-100) and 58.53% (without Triton X-100), while maximum recoveries were 100.96% (with Triton X-100) and 99.50% (without Triton X-100). The statistical analysis indicated that the percent recoveries of metals obtained by digestion of lipsticks with acids enhanced with Triton X-100 were significantly higher than similar digestions without Triton X-100. The 95% Confidence Interval suggests that if a large number of samples is taken, 95% of the time the percentage recoveries fall in the interval ( 81.61, 94.87%) with Triton X-100 and (74.48, 88.76%) without Triton X-100. The statistical comparison as displayed in Table 9 for lipsticks, showed that the percentage recovery for the heavy metals of interest digested using Method A-1 (99.85 ± 1.32%) > Method C-1 (89.51 ± 7.67%) > Method B-1(75.37 ± 13.46%). The minimum percentage recoveries of Method A-1, B-1 and C-1 were 97.41%, 60.90% and 80.50% respectively, while maximum concentrations were 100.96%, 96.24% and 99.54% respectively. The 95% Confidence Interval suggests that if a large number of samples were taken, 95% of the time the percentage recoveries will fall within the interval of Method A-1 (98.46, 101.24%), Method B-1 (61.24, 89.50%) and Method C-1 (81.46, 97.57%). This indicates that the highest percentage recoveries will be obtained if method A-1 is utilized to digest lipstick samples (Table 12).

**Table 7:** Average Percent Recoveries (%) for spiked samples of Lipsticks.

	Elements	Acid Digestion Methods		
		A-1 (HNO_3(aq)_ + H_2_O_2(aq)_)	B-1 (HCl_(aq)_ + HNO_3(aq)_	C-1 (HNO_3(aq)_ + HCl_(aq)_ H_2_O_2(aq)_)
With Triton X-100	As	100.96	73.10	80.50
	Cd	99.63	67.27	91.46
	Cr	100.57	60.90	96.54
	Pb	99.71	67.82	82.31
	Hg	97.41	96.24	99.54
	Ni	100.85	86.93	86.75

		A-2 (HNO_3(aq)_ + H_2_O_2(aq)_)	B-2 (HCl_(aq)_ + HNO_3(aq)_	C-2 (HNO_3(aq)_ + HCl_(aq)_ H_2_O_2(aq)_)

Without Triton X-100	As	99.50	74.50	79.85
	Cd	91.88	65.84	88.91
	Cr	81.13	58.53	87.58
	Pb	85.92	62.72	69.58
	Hg	89.11	83.64	79.72
	Ni	97.92	84.24	81.07

**Table 8:** Statistical analysis of% recovery data derived from spiked Lipsticks analyzed using Triton x100 and without Triton x-100.

	N	Mean	Std. Deviation	Std. Error	95% Confidence Interval for Mean		Minimum	Maximum
					Lower Bound	Upper Bound		
With Triton x-100	18	88.2494	13.3319	3.1424	81.6196	94.8793	60.90	100.96
Without Triton x-100	18	80.4239	11.9491	2.8164	74.4817	86.3660	58.53	99.50

**Table 9:** Comparison of Mean Percentage Recoveries for Method A-1, B-1 and C-1 for Lipstick.

Digest	N	Mean	Std. Deviation	Std. Error	95% Confidence Interval for Mean		Minimum	Maximum
					Lower Bound	Upper Bound		
A-1	6	99.8550	1.3245	0.5407	98.4650	101.2450	97.41	100.96
B-1	6	75.3767	13.4635	5.4965	61.2475	89.5058	60.90	96.24
C-1	6	89.5167	7.6741	3.1329	81.4632	97.5702	80.50	99.54

**Table 10:** ANOVA Comparison of Percent Recoveries between and within groups for Lipsticks.

	Sum of Squares	Df	Mean Square	F	Sig.
Between Groups	1812.019	2	906.010	11.236	0.001
Within Groups	1209.570	15	80.638		
Total	3021.589	17

**Table 11:** Test of Homogeneity of Variances for the Heavy Metal Recoveries of Lipsticks.

Levene Statistic	Df1	Df2	Sig.
0.488	1	34	0.489

**Table 12:** Average Percent Recoveries (%) for the analysis of spiked samples of face powders.

	Elements	Acid Digestion Methods		
		A-1 (HNO_3(aq)_ + H_2_O_2(aq)_)	B-1 (HCl_(aq)_ + HNO_3(aq)_	C-1 (HNO_3(aq)_ + HCl_(aq)_ H_2_O_2(aq)_)
With Triton X-100	As	98.50	65.25	82.17
	Cd	92.61	83.27	87.50
	Cr	95.12	77.11	93.54
	Pb	95.12	69.16	74.39
	Hg	97.61	83.44	92.16
	Ni	97.44	63.91	86.75

		A-2 (HNO_3(aq)_ + H_2_O_2(aq)_)	B-2 (HCl_(aq)_ + HNO_3(aq)_	C-2 (HNO_3(aq)_ + HCl_(aq)_ H_2_O_2(aq)_)

Without Triton X-100	As	87.57	63.71	81.85
	Cd	89.43	77.22	85.83
	Cr	83.07	66.35	84.21
	Pb	79.91	50.91	69.02
	Hg	88.36	81.09	79.73
	Ni	88.52	62.08	84.17

In Table 15, the Analysis of Variances (ANOVA) test suggests that, at the 5% level, there are no statistically significant differences among the mean recoveries of the powders, since *p* = 0.056 (i.e. *p* > 0.05). The assumption of equal population variance is met by the data as shown in Table 16, since *p* = 0.782 (i.e., *p* > 0.05). The statistical comparison displayed in Table 13 for powders, showed that the percentage recovery for the heavy metals of interest digested with the addition of Triton X-100 were higher (85.24 ± 11.28%) than that recorded without the use of Triton X-100 (77.94 ± 10.96%). The minimum recoveries were 63.91% (with Triton X-100) and 50.91% (without Triton X-100), while maximum recoveries were 98.50% (with Triton X-100) and 89.50% (without Triton X-100). The 95% Confidence Interval suggests that if a large number of samples are taken, 95% of the time the percentage recoveries will fall within the interval ( 79.66, 90.89%) with Triton X-100 and (72.49, 83.39%) without Triton X-100. The statistical comparison as displayed in Table 14 for powders, showed that the percentage recovery for the heavy metals of interest digested using Method A-1 (96.06 ± 2.18%) > Method C-1 (86.08 ± 7.02%) > Method B-1(73.69 ± 8.78%). The minimum percentage recoveries of Method A-1, B-1 and C-1 were 92.61%, 63.91% and 74.39% respectively, while maximum recoveries were 98.50%, 83.44% and 93.54%, respectively. The 95% Confidence Interval suggests that if a large number of samples were taken, 95% of the time the percentage recoveries will fall within the interval of Method A-1 (93.77, 98.36%), Method B-1 (64.47, 82.91%) and Method C-1 (78.70, 93.46%). This indicates that the highest percentage recoveries will be obtained if method A-1 is utilized to digest powdered samples.

**Table 13:** Statistical analysis of% recovery data derived from spiked Face Powders analyzed using Triton x100 and without Triton x-100.

	N	Mean	Std. Deviation	Std. Error	95% Confidence Interval for Mean		Minimum	Maximum
					Lower Bound	Upper Bound		
With Triton x-100	18	85.2806	11.2847	2.6598	79.6688	90.8923	63.91	98.50
Without Triton x-100	18	77.9450	10.9629	2.5839	72.4932	83.3968	50.91	89.43

**Table 14:** Comparison of Mean% Recoveries for Method A-1, B-1 and C-1 for Powders.

	N	Mean	Std. Deviation	Std. Error	95% Confidence Interval for Mean		Minimum	Maximum
					Lower Bound	Upper Bound		
A-1	6	96.0667	2.1876	0.8931	93.7708	98.3625	92.61	98.50
B-1	6	73.6900	8.7856	3.5867	64.4700	82.9100	63.91	83.44
C-1	6	86.0850	7.0289	2.8695	78.7086	93.4614	74.39	93.54

**Table 15:** ANOVA Comparison of Percent Recoveries between and within groups for Powders.

	Sum of Squares	Df	Mean Square	F	Sig.
Between Groups	484.293	1	484.293	3.913	0.056
Within Groups	4208.035	34	123.766		
Total	4692.329	35

**Table 16:** Test of Homogeneity of Variances for the Heavy Metal Recoveries of Powders.

Levene Statistic	Df1	Df2	Sig.
0.078	1	34	0.782

For both lipsticks and face powders, digestion Method A-1 (Nitric Acid and Hydrogen Peroxide with Triton X-100 at 95 °C for 3 h) proved to be the most efficient at extracting the heavy metals form the matrices. With percentage recoveries, ranging from 97.41% to 100.9% for lipsticks and 92.61% to 98.56% for powders there appears to be insignificant losses of heavy metals during acid digestion.

The surfactant properties of Triton X-100 enhances the efficiency of acid digestion by effectively dispersing the hydrophobic sample within the aqueous acid. The homogenous emulsion formed allows for greater contact between the digesting media and the sample, which improves the digestion process.

## Conclusion

Acid digestion is one of the most time intense and labor rigorous step of heavy metal analysis. Considering the large quantity of cosmetic samples to be analyzed, it was necessary to determine the most efficient method that gave the best heavy metal recoveries, reduce analysis time and guarantees cost effectiveness.

At the 5% level of significance, the results for Acid digestion using Method A-1 (Nitric Acid and Hydrogen Peroxide with Triton X-100 at 95 °C for 3 h) were validated and was the most efficient and suitable digestion method in this study. The addition of Triton X-100 gave superior results when compared to digestions without Triton X-100.

## Declaration of Competing Interest

The Authors confirm that there are no conflicts of interest.

## References

[1] Zakaria A., Ho Y.B. (2015). Heavy metals contamination in lipsticks and their associated health risks to lipstick consumers. Regulat. Toxicol. Pharmacol..

[2] Batista E.F., Augusto A.d.S., Pereira-Filho E.R. (2014). Determination of Cd, Co, Cr, Cu, Ni and Pb in cosmetics samples using a simple method for sample preparation. R. Soc. Chem..

[3] Khalid A., Bukhari I.H., Riaz M., Rehman G., Ain Q., Bokhari T.H., Rasool N., Zubair M., Munir S. (2013). Determination of lead, cadmium, chromium, and nickel in different brands of lipsticks. Int. J. Biol. Pharm. Allied. Sci..

[4] Salama A.K. (2016). Assessment of metals in cosmetics commonly used in Saudi Arabia. Environ. Monit. Assess.

[5] Sani A., Gaya M.B., Abubakar F.A. (2016). Determination of some heavy metals in selected cosmetic products sold in kano metropolis, Nigeria. Toxicol. Rep..

[6] Omenka S.S., Adeyi A.A. (2016). Heavy metal content of selected personal care products (PCPs) available in Ibadan, Nigeria and their toxic effects. Toxicol. Rep..

[7] Iwegbue C.M.A., Bassey F.I., Obi G., Tesi G.O., Martincigh B.S. (2016). Concentrations and exposure risks of some metals in facial cosmetics in Nigeria. Toxicol. Rep..

[8] Zhong Z., Li G., Luo J., Chen W., Liu L., He P., Luo Z. (2015). Proficiency testing for determination of lead and arsenic in cosmetics: comparison of analytical procedures and evaluation of laboratory performances. Anal. Methods.

[9] Bass D.A., Hickock D., Quig D., Urek K. (2001). Trace element analysis in hair: factors determining accuracy, precision, and reliability. Alternative Med. Rev..

[10] da Silva J.B., Borges D.L., da Veiga M.A., Curtius A.J., Welz B. (2003). Determination of cadmium in biological samples solubilized with tetramethylammonium hydroxide by electrothermal atomic absorption spectrometry, using ruthenium as permanent modifier. Talanta..

[11] Batista B.L., Rodrigues J.L., Nunes J.A., Tormen L., Curtius A.J., Barbosa F.J. (2003). Simultaneous determination of Cd, Cu, Mn, Ni, Pb and Zn in nail samples by inductively coupled plasma mass spectrometry (ICP-MS) after tetramethylammonium hydroxidesolubilization at room temperature: comparison with ETAAS. Talanta..

[12] Ishak I., Rosli F.D., Mohamed J., Ismail M.F.M. (2015). Comparison of digestion method for the determination of trace elements and heavy metals in human hair and nails. Malays. J. Med. Sci..

[13] Adeloju S.B. (1989). Comparison of some wet digestion and dry ashing methods for voltammetric trace element analysis. R. Soc. Chem..

[14] Mohammed E., Mohammed T., Mohammed A. (2017). Optimization of an acid digestion procedure for the determination of Hg, As, Sb, Pb and Cd in fish muscle tissue. Methods X.

[15] Matusiewicz H. (2003). Wet digestion methods. Comprehensive Anal. Chem..

[16] Xiao J. (2004). Sample Preparation and Heavy Metal Determination by Atomic Spectroscopy.

[17] Ullah H., Noreen S., Fozia A.Rehman, Waseem A., Zubair S., Adnan M., Ahmad I. (2017). Comparative study of heavy metals content in cosmetic products of different countries marketed in Khyber Pakhtunkhwa, Pakistan. Arab. J. Chem..

[18] El-Aziz R.A., Abbassy M.M., Hosny G. (2017). Health risk assessment of some heavy metals in cosmetics in common use. Sci-Afric J. Sci. Issues.

[19] Sainio E.-.L., Jolanki R., Hakala E., Kanerva L. (2000). Metals and arsenic in eye shadows. Contact Derm..

[20] Hepp N.M., Mindak W.R., Gasper J.W., Thompson C.B., Barrows J.N. (2014). Survey of cosmetics for arsenic, cadmium, chromium, cobalt, lead, mercury, and nickel content. J. Cosmet. Sci..

[21] J.K. Nduka, I.O. Odiba, E.I. Aghoghome, N.L. Umedum, M.J. Nwosu, Evaluation of harmful substances and health risk assessment of mercury and arsenic in cosmetic brands in Nigeria, 2016 8(1) (2016).

[22] Kingston H.M., Walter P.J. (1986). Microwave energy for acid decomposition at elevated temperatures and pressures using biological and botanical samples. Anal. Chem..

[23] B.J. Alloway, Heavy Metals in soils: Trace Metals and Metalloids in Soils and Their Bioavailability, Springer Science & Business Media2012.

[24] Mohammed E., Mohammed T., Mohammed A. (2018). Optimization of instrument conditions for the analysis for mercury, arsenic, antimony and selenium by atomic absorption spectroscopy. MethodsX.

[25] Prichard E., Barwick V. (2007). Quality Assurance in Analytical Chemistry.

[26] (1997). The Harzadous Materials Training and Research Institute, Site Characterization Sampling and Analysis.

[27] Harris D.C. (2010). Quantitative Chemical Analysis.

[28] Shrivastava A., Gupta V.B. (2011). Methods for the determination of limit of detection and limit of quantitation of the analytical methods. Chronicles Young Sci..

[29] Miller J.N., Miller J.C. (2010). Statistics and Chemometrics for Analytical Chemistry.

